# Recent Advances in Polymer Nanocomposites Based on Polyethylene and Polyvinylchloride for Power Cables

**DOI:** 10.3390/ma14010066

**Published:** 2020-12-25

**Authors:** Diaa-Eldin A. Mansour, Nagat M. K. Abdel-Gawad, Adel Z. El Dein, Hanaa M. Ahmed, Mohamed M. F. Darwish, Matti Lehtonen

**Affiliations:** 1 High Voltage and Superconductivity Laboratory, Department of Electrical Power and Machines Engineering, Faculty of Engineering, Tanta University, Tanta 31511, Egypt; mansour@f-eng.tanta.edu.eg; 2 Department of Electrical Engineering, Faculty of Engineering at Shoubra, Benha University, Cairo 11629, Egypt; nagah.abdelgwad@feng.bu.edu.eg; 3 Department of High Voltage Networks, Faculty of Energy Engineering, Aswan University, Aswan 81528, Egypt; adelzein@energy.aswu.edu.eg; 4 Department of Mathematics and Engineering Physics, Faculty of Engineering at Shoubra, Benha University, Cairo 11629, Egypt; hana.ahmed@feng.bu.edu.eg; 5 Department of Electrical Engineering and Automation, School of Electrical Engineering, Aalto University, Espoo 02150, Finland; matti.lehtonen@aalto.fi

**Keywords:** dielectric properties, polymer nanocomposites, polyethylene, polyvinylchloride, underground cables

## Abstract

Polymer nanocomposites used in underground cables have been of great interest to researchers over the past 10 years. Their preparation and the dispersion of the nanoparticles through the polymer host matrix are the key factors leading to their enhanced dielectric properties. Their important dielectric properties are breakdown strength, permittivity, conductivity, dielectric loss, space charge accumulation, tracking, and erosion, and partial discharge. An overview of recent advances in polymer nanocomposites based on LDPE, HDPE, XLPE, and PVC is presented, focusing on their preparation and electrical properties.

## 1. Introduction

Due to their reliability, availability, ease of fabrication, and low cost, polymers have been widely used as electrical insulating materials for underground cables since the early 20th century [1]. In general, polymers are defined as macromolecules consisting of many repeating units called monomers. On the basis of intermolecular forces acting within their chains, polymers can be classified into three main classes: elastomers, such as natural rubber and polyurethane; thermosets, such as epoxy resins; and thermoplastics, such as polyethylene (PE) and polyvinyl chloride (PVC). PE and PVC are considered the most commonly synthetic polymers used as electrical insulating materials due to their low permittivity and high electrical breakdown strength [2]. PE can be found in several forms: high-density polyethylene (HDPE), low-density polyethylene (LDPE), linear low-density polyethylene (LLDPE), and cross-linked polyethylene (XLPE) [1,2].

The commercial homo-polymer form of HDPE (with density ranged from 0.941–0.959 g/cm^3^) is a linear polymer that contains 94% crystalline phase and 6% amorphous phase [3]. Its low chemical reactivity, stiffness, impermeability, thermal stability, and moisture resistivity reveal HDPE to be applicable in electrical insulation [4,5,6].

Compared to HDPE, LDPE with lower density (0.910–0.925 g/cm^3^), lower chemical reactivity, and lower tensile strength, due to the presence of short and long branched chains [7], is widely used in the fabrication of cables for high-voltage direct-current (HVDC). Further, LDPE can withstand a temperature of 95 °C for a short time and has a higher resistance to dilute, concentrated acids, and bases than many other organic compounds. Nevertheless, it has poor resistance to halogenated hydrocarbons [8].

In addition to LDPE, LLDPE was also used in the insulation of wires and cables for low, medium, and high voltage applications. It was first commercialized in the 1970s by Union Carbide and Dow Chemical. The main difference between LLDPE and LDPE is that the former has a narrow molecular weight distribution and no long chain branching, therefore it has higher tensile strength and puncture resistance than LDPE [7]. Its density (0.926–0.940 g/cm^3^) is higher than that of LDPE [9], and since it is a saturated hydrocarbon, like polyethylene, it is generally unreactive; it resists alcohols, alkaline solutions, weak organic or inorganic acids, and saline solutions [7,10,11].

Due to the drawbacks of PE; the limitation of the maximum operating temperature (70 °C) and its deterioration due to the absence of antioxidants, a new type of PE was introduced by crosslinking the PE in order to improve its thermal and aging stability [12,13]. Cross-linked polyethylene (XLPE) is typically a modified HDPE, with permanently chemically linked polymeric chains. The crosslinking proceeds through the chemical reaction of polymeric chains with materials possessing multifunctional groups, which join the polymeric chains together through chemical bonds, forming a three-dimensional dense network, thermosets polymers, with totally different properties, such as their chemical structure and resistance, and their mechanical performance (environmental stress, toughness, abrasion and crack resistance), and chemical resistance [14,15,16]. Depending on the degree of crosslinking, XLPE has been used in several applications, e.g., electrical insulation, pipes, and packaging [12,13,17,18].

In addition to polyethylene and its different forms, polyvinylchloride (PVC) is another thermoplastic polymer used in electrical insulation. It has excellent fire resistance due to the presence of chlorine, which made it one of the most widely produced synthetic polymers [2], with multiple applications in pipelines, ducts, and electrical insulation. PVC is produced in a rigid or flexible form; the latter being preferred for electrical insulation. Despite high hardness and good mechanical properties, PVC starts to decompose at 140 °C, however, its thermal properties may be improved by the addition of heat stabilizers [19,20].

Recently, nanodielectrics have been developed for many applications. Nanodielectrics are polymer dielectrics filled with a certain weight percent (wt %) of inorganic nanoparticles homogeneously dispersed in the polymeric matrix. Such materials, containing low concentrations of nanoparticles, exhibit promising enhancement of their electrical, mechanical, and thermal properties compared to conventional materials [21,22,23]. This enhancement is largely due to the large surface area per unit volume of the nanoparticles. Nanodielectrics can also act as multifunctional materials: controlling the elevation and cryogenic in temperature, controlling the thermal conductivity for the insulators, and increasing the energy density of capacitor systems. This is because of the simultaneous enhancement of the above-mentioned properties [24], and consequently, they satisfy in large measure the current needs of the electrical power industry. Some examples are epoxy, polyethylene, and polypropylene nanodielectrics [25].

**Figure 1:**
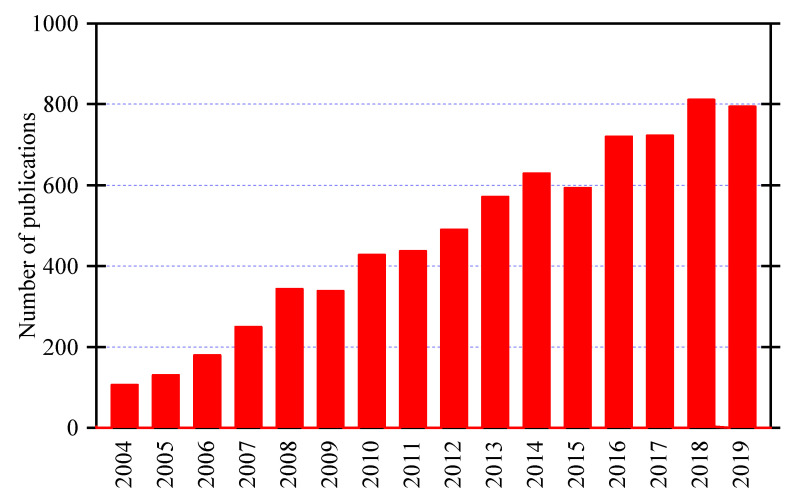
Annual publications on nanocomposites based on polyethylene and polyvinylchloride according to Web of Science database.

Polymer nanocomposites used in underground cables have been of great interest to researchers over the past years [26,27,28]. Figure 1 shows the growth of research articles in nanocomposites based on polyethylene and polyvinylchloride in the last 15 years according to Web of Science database. It is clear that there is an increasing trend in the number of research articles covering this topic, which indicates the importance of this topic for the academy and industry. The preparation and the dispersion of the nanoparticles through the polymer host matrix are the key factors leading to the enhanced dielectric properties of polymer nanocomposites used in underground cables. Their important dielectric properties are breakdown strength, dielectric loss, space charge accumulation, tracking and erosion, and partial discharge. We discuss these properties in this article.

## 2. Preparation of Polymer Nanocomposites

The type, size, and surface morphology of the nanoparticles are the critical factors in enhancing the properties of polymer nanocomposites. Nanoparticles may be obtained through various preparation techniques, e.g., sol-gel [29], chemical precipitation [30,31], and electrode deposition [32,33]. The selection of the appropriate method depends on the physical and chemical characteristics of the host polymer matrices. The most common nanoparticles dispersed in insulating polymeric hosts are clay, silica (SiO_2_), titania (TiO_2_), alumina (Al_2_O_3_), and other metal oxides. The benefits of nanoparticle dispersion are realized when the dispersion is uniform, with little particle agglomeration.

The overall physical and chemical properties of polymer nanocomposites are controlled by the properties of both nanoparticles and polymers, the interactions between them, and the polymer/nanoparticle interface [34]. It is reported that at this interface there is a fraction of polymer is confined and grafted at the highly surface-area nanoparticles, thus, there is a gradual transition of the physical and the chemical properties from “bulk” matrix-controlled features to interphase or “surface” dominated characteristics [35]. Therefore, the unique structure of this special region has dominated influence on the electrical and mechanical properties of the nanocomposite materials, and its influence becomes more significant in nanocomposites with larger interfacial areas compared with microcomposites [36]. In order to observe such an influence, a uniform dispersion of the nanoparticles through the polymer matrix is mandatory [35].

Unfortunately, the small size and the high surface area-to-volume ratio of nanoparticles increases their tendency to agglomerate within the polymeric matrix, introducing additional challenges compared to the preparation of microcomposites [37,38]. Agglomerations of nanoparticles are considered weak points at which destructive processes, such as electrical tree initiation or mechanical crack propagation, can occur, resulting in deterioration of the electrical, mechanical, and thermal properties of the nanocomposite.

The dispersion of nanoparticles within the polymer can be improved through physical approaches, chemical modifications, or both [39]. Physical approaches include mechanical mixing and ultrasonic agitation. The polymer and nanoparticles are mixed together with stabilizing agents before compounding. In this way, nanoparticles can be separated from each other without changing the chemical nature of the nanoparticles or the host polymer matrix [40]. However, nanocomposites obtained by this approach suffer from poor adhesion of the nanoparticles to the polymer matrix, due to incompatibility between hydrophilic and hydrophobic constituents preventing optimal dispersion of the nanoparticles. To prevent this, chemical modification using compatibilizers, e.g., coupling agents, is used in order to reduce the surface energy of the nanoparticles [41,42,43,44]. The best results are obtained through chemical modification of the nanoparticle surfaces, allowing the nanoparticles to graft to the polymer chains [45,46,47].

**Figure 2:**
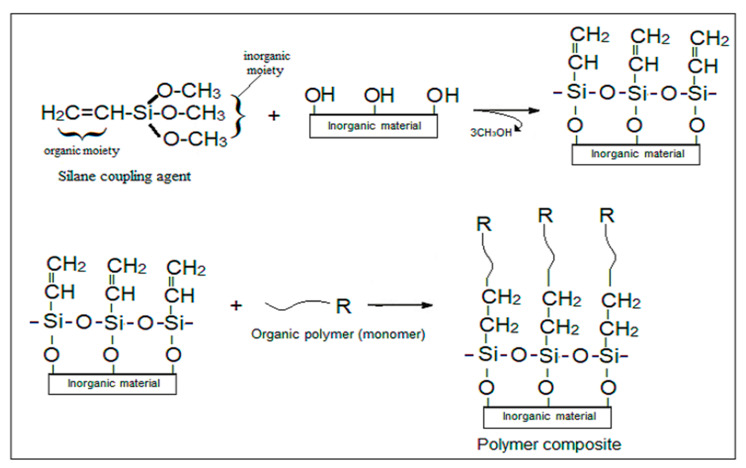
Synthesis of polymer composite upon functionalization of inorganic materials (such as metal oxides nanoparticles) with silane coupling.

Materials such as aluminates, silicates, and borates are often involved in linking dissimilar surfaces. The most commonly used coupling agents in polymer nanocomposites are silanes which form durable chemical bonds between organic polymers and inorganic additives in a such way, as shown in Figure 2 [48,49]. The general formula of a silane coupling agent, R–(CH_2_)_n_–Si–X_3_, with two functional groups, namely the inorganic moiety X, (such as alkoxy, acyloxy, halogen, and amino groups), and the organic moiety R (typically amino, epoxy, and vinyl groups). Silane coupling agents may form a covalent bond directly with the finished polymer (thermoplastic polymers) or may be copolymerized with the monomer (thermoset polymers) [50].

The effect of nanoparticle surface modification by a silane coupling agent has been investigated in detail for SiO_2_/XLPE nanocomposites [51]. The dielectric constant decreased for nano-sized modified SiO_2_, compared to micro-sized modified SiO_2_. This observation was explained in terms of the particle surface curvature, which is high for a nanoscale diameter, resulting in a decrease in the amount of hydrogen bonding between the silanol groups. Accordingly, the interfacial polarization and the nanocomposite dielectric constant are reduced.

There are three different methods of preparing polymer nanocomposites [52], namely in-situ polymerization, melt blending, and polymer dissolution mixing. The latter two methods are used to prepare polymer nanocomposites for use in underground cables. In the melt blending method, the polymer material is heated above its melting temperature, e.g., 135 °C for LDPE [53], and nanoparticles are then mixed with and dispersed into the melted polymer. Finally, the mixture is extruded, pressed into sheets of the required thickness, and allowed to cool to room temperature. In the polymer dissolution mixing method a suitable solvent for the host polymeric material is added, e.g., tetrahydrofuran solvent for PVC [54] and xylene solvent for LDPE [55]. Nanoparticles are then added directly to the solution or dispersed in the solvent before being added to the solution. In order to reduce the time required to obtain a homogeneous mixture and uniform dispersion of the nanoparticles, magnetic stirring and ultrasonic homogenization may be used. The resulting liquid is poured into a mold and the solvent is allowed to volatilize, either in the air over several days or in a vacuum oven for a few hours.

## 3. Dielectric Properties of Polymer Nanocomposites

Polymer nanocomposites exhibit enhanced dielectric properties compared to conventional polymeric materials. We consider now dielectric breakdown strength, permittivity, conductivity, dielectric loss, space charge behavior, and partial discharge (PD) resistance.

### 3.1. AC Breakdown Strength

**Figure 3:**
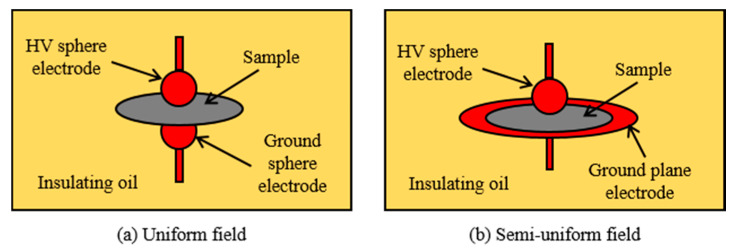
Electrode configurations used for measuring breakdown strength; (**a**) uniform field and (**b**) semi-uniform field.

Many experimental studies of the breakdown strength of nanocomposites have been reported [56,57,58,59,60,61,62,63,64,65]. In most cases, the measurements were carried out under a uniform electric field or semi-uniform electric field using sphere-to-sphere electrode or sphere-to-plane electrode, respectively, as shown in Figure 3. For both electrode configurations, they are immersed in oil in order to prevent surface flashover. The frequency of the applied AC voltage is the power frequency, either 50 Hz or 60 Hz.

**Figure 4:**
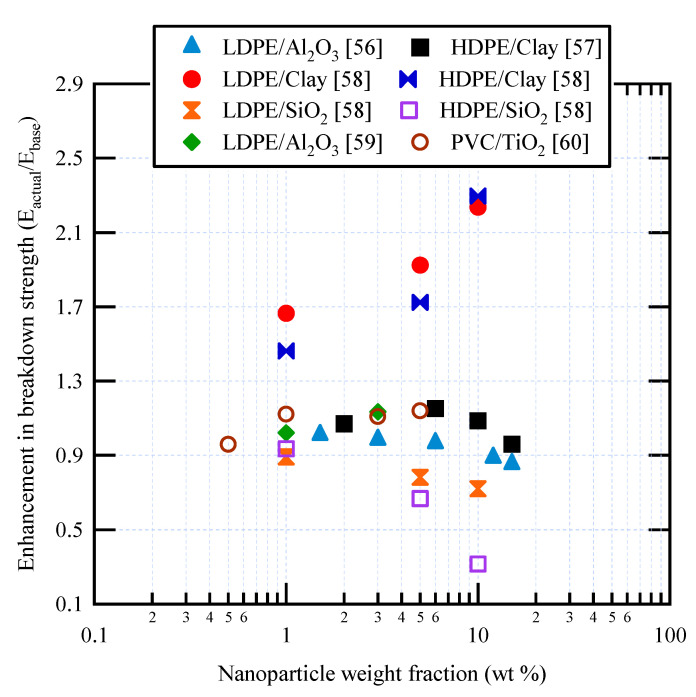
Some published data for AC breakdown strength in various polymer nanocomposites.

For AC breakdown strength of polymer nanocomposites based on PE and PVC, Table 1 summarizes the key findings obtained in the literature. Also, Figure 4 shows some experimental data for AC breakdown strength as a function of nanoparticle weight fraction. Most enhancements do not exceed 20%, i.e., E_actual_/E_base_ < 1.2. There are some differences between the results obtained by different authors for nominally identical nanocomposites. Thus, for HDPE/nanoclay, at 10 wt % nanoparticle fraction, an increase of approximately 130% was reported in [58], but only about 10% in [57]. On the other hand, for LDPE/Al_2_O_3_, very small increases were reported in [56,59] for small nanoparticle fractions. The decreases in AC breakdown strength might be due to the use of untreated SiO_2_ nanoparticles, which could have limited compatibility with the polymer matrix.

There are two main factors responsible for the variability seen in these results. The first is the varying extent of nanoparticle agglomeration due to differences in preparation procedure, choice of coupling agent, and choice of surface treatment. Even small differences can cause significant differences in dielectric properties [66]. Nanoparticle agglomerates less than 3 µm in diameter were found in LDPE/Al_2_O_3_ nanocomposites, and no enhancement was obtained in the breakdown strength [56]; increasing nanoparticle agglomerate size resulted in a reduction in breakdown strength relative to the host LDPE matrix. On the other hand, LDPE/Al_2_O_3_ nanocomposites [59] exhibited a slight enhancement in AC breakdown strength despite the use of nanoparticles of the same type and almost the same size as specified in [56]. Scanning electron micrographs presented in both publications show that nanoparticle agglomeration was smaller in [59] than in [56], probably due to the use of ultrasonic bath mixing in the latter.

The second factor causing variability in the results is the breakdown strength of the base material itself. Thus in [58], the host polymer matrices (LDPE and HDPE) had low breakdown strength, leading to greater enhancements following the addition of nanoparticles.

In addition to the effect of the type and weight fraction of nanoparticles on dielectric breakdown strength, several studies have highlighted the important effects of nanoparticle surface modification. Huang et al. [43] showed that SiO_2_ nanofillers modified by octasilane slightly increased the AC breakdown strength of LLDPE; similarly, functionalized fumed SiO_2_ nanofillers slightly increased the AC breakdown strength of XLPE [61]. However, modification of TiO_2_ nanoparticles [62] and SiO_2_ nanoparticles [63] by polyhedral oligomeric silsesquioxane increased the AC breakdown strength of LDPE by 9% and 7%, respectively. Surface modification of TiO_2_ nanoparticles by vinyl silane coupling also caused an 11% increase in the AC breakdown strength of PVC/TiO_2_ nanocomposites [60].

### 3.2. DC Breakdown Strength

**Figure 5:**
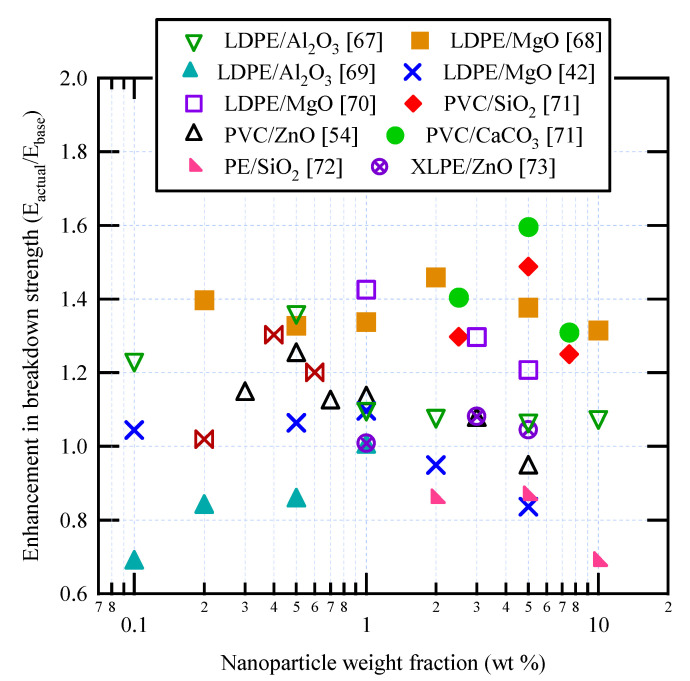
Some published data for DC breakdown strength in polymer nanocomposites.

The electrode configuration used for measuring DC breakdown strength follows the same configurations depicted in Figure 3. Table 2 summarizes the key findings obtained in the literature for DC breakdown strength of polymer nanocomposites based on PE and PVC, while Figure 5 shows some experimental data for DC breakdown strength as a function of nanoparticle weight fraction [42,54,67,68,69,70,71,72,73]. There are considerable differences between the results obtained by different authors for nominally identical nanocomposites, e.g., LDPE/Al_2_O_3_ at 0.5% nanoparticle wt fraction reported in [67,69], and LDPE/MgO at 5% nanoparticle wt fraction reported in [42,68,70]. These differences have been attributed to the high dependence of DC breakdown strength on space charge behavior, which in turn is sensitive to preparation processes and purity of materials [74,75]. Dispersion of SiO_2_ nanoparticles in PE resulted in a reduction in the DC breakdown strength over the nanoparticle wt fraction range 1–10% [72], similar to the AC breakdown strength behavior results reported in [58]. However, SiO_2_ nanoparticles in PVC greatly increased the DC breakdown strength [71]. The decrease in DC breakdown strength could be due to the use of untreated SiO_2_ nanoparticles [58], or inappropriate type/chain length of coupling agent [72]. Effective surface modification of SiO_2_ nanoparticles could increase the DC breakdown strength by as much as 30% [74].

### 3.3. Impulse Breakdown Strength

**Figure 6:**
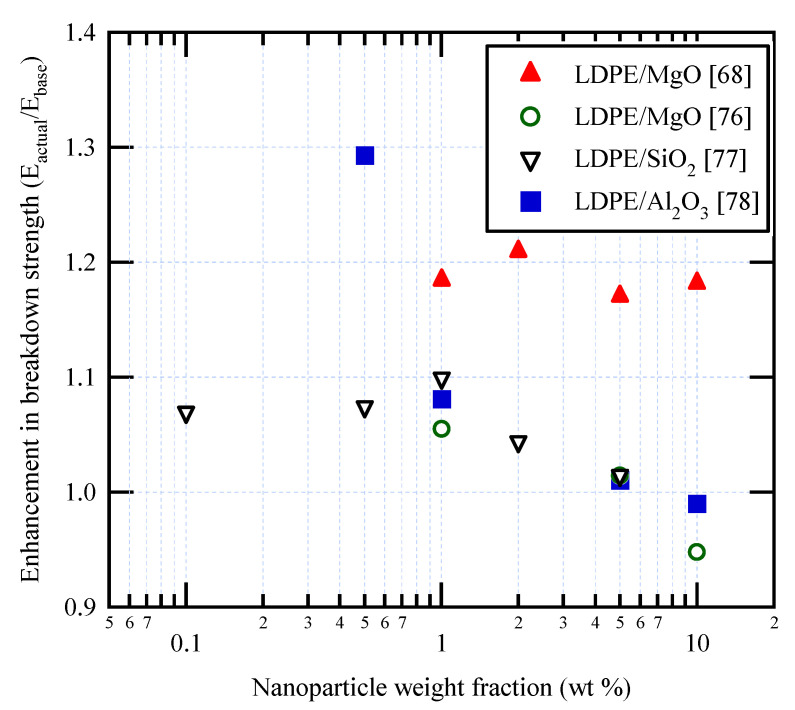
Some published data for impulse breakdown strength in polymer nanocomposites.

Few researchers have studied the impulse breakdown strength of nanocomposites [68,76,77,78]. In this regard, Table 3 and Figure 6 summarize the key findings obtained in the literature. As shown in Figure 6, the impulse breakdown strength tends to decrease with increasing nanoparticle concentration above approximately 1%, with the exception of the [68] data. The percentage increases also tend to be smaller than those under AC and DC [77]. The reasons for the differences between impulse and AC/DC breakdown strength are still unclear. It seems that impulse breakdown strength for PVC nanocomposites is not available in the open literature and needs to be studied.

### 3.4. Breakdown Mechanisms

**Figure 7:**
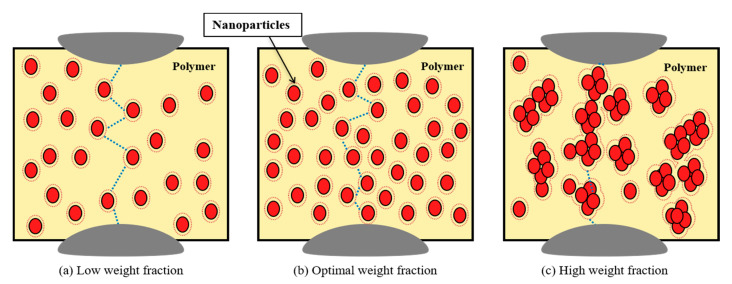
Effect of distribution and agglomeration of nanoparticles on breakdown strength in polymer nanocomposites; (**a**) low weight fraction, (**b**) optimal weight fraction, and (**c**) high weight fraction.

Two main mechanisms have been proposed to explain the mostly increase in the breakdown strength of polymer nanocomposites, relative to the host polymer matrix. The first emphasizes the role of nanoparticles and the nanoparticle/polymer interfaces in forming barriers capable of preventing the growth of discharge channels [79,80]. Other studies have emphasized the role of the interfacial regions in creating a rigid barrier around the nanoparticles [81,82], resulting in increased charge carrier energy loss. The second mechanism emphasizes the role of deep traps in capturing charge carriers, thereby decreasing their mobility and energy [67,83]. Thus, both mechanisms depend on the total surface provided by nanoparticles. For most cases depicted above, there is an optimal weight fraction, above which a decrement in breakdown strength is observed. This is can be attributed to the effect of distribution and agglomeration of nanoparticles as shown in Figure 7. With a low weight fraction of nanoparticles in Figure 7a, they are distributed uniformly all over the polymer sample leading to an elongation in the discharge path and an existence of multiple trapping sites for charge carriers, thereby enhancing breakdown strength. Increasing the weight fraction of nanoparticles in Figure 7b provides a larger surface area with further enhancement in breakdown strength. At high nanoparticle loadings in Figure 7c, the agglomeration of nanoparticles and the overlap between interfacial regions result in transport paths for charge carriers, and consequently a reduction in breakdown strength.

### 3.5. Permittivity, Conductivity, and Dielectric Loss

Considerable research has been reported on the permittivity, conductivity, and dielectric loss of nanodielectrics in general [81,84,85,86,87]. However, very few corresponding data are available for PE and PVC based nanodielectrics. The permittivity considered in the literature refers to relative permittivity or dielectric constant. Table 4 summarizes most of the key results obtained regarding the dielectric properties of polymer nanocomposites based on PE and PVC.

The permittivity of LDPE has been reported to increase with the addition of 0.015% volume fraction of graphene oxide [88]. Increased permittivity has also been observed with the addition of multiwall carbon nanotubes (MWCNT) [89]; at a volume fraction 0.08% of MWCNT, the permittivity was 100, compared with 2.2 for LDPE. As reported in [69], the permittivity of an LDPE/Al_2_O_3_ nanocomposite containing Al_2_O_3_ nanoparticles functionalized through vinyl silane coupling was lower than that of neat LDPE. The authors suggested that the functionalized Al_2_O_3_ nanoparticles lowered the permittivity by inhibiting the movement of charge carriers. The permittivity was independent of frequency over the frequency range 100 Hz–1 MHz, suggesting electrode polarization originating in blocking of charge carriers at the electrode/LDPE interfaces [90]. A similar trend was observed in PVC/functionalized TiO_2_ nanocomposites [60], in which the permittivity decreased by about 43% relative to neat PVC, and by about 33% relative to PVC/unmodified TiO_2_ nanocomposites. It appears that the permittivity increases/decreases depending on the type and the size of the nanoparticles, and on the surface modification coupling agent.

In relation to conductivity, Wang et al. [67] showed that the DC volume resistivity of LDPE/Al_2_O_3_ nanocomposites increased with increasing Al_2_O_3_ weight fraction, the increase being a factor of about 10 at 0.5 wt % loading, but limited at higher loading. Modified Al_2_O_3_ nanoparticles with vinyl silane coupling also increased the DC volume resistivity to about three times that of neat LDPE [69]. On the other hand, uncoated MgO and Al_2_O_3_ nanoparticles caused a reduction of approximately 98% in the conductivity of LDPE nanocomposites at a weight fraction of 3% [91]. However, Al_2_O_3_ nanoparticles coated with silanes of terminal alkyl groups of different lengths caused a similar decrease in the conductivity of LDPE nanocomposites, but at a loading of 1 wt % [92].

Field-dependent DC conductivity has been reported in LDPE/graphene oxide nanocomposites [88]. Below approximately 4 kV/mm the nanocomposites showed lower conductivity than neat LDPE. The authors suggested that the LDPE/nanoparticle interfaces acted as physical barriers to current flow, thereby decreasing conductivity. However, above 4 kV/mm the nanocomposites showed conductivity values higher than that of LDPE. A much greater increase in the conductivity of these nanocomposites with increasing temperature was observed than for LDPE; this was attributed to the thermal energy gained by charge carriers at higher temperatures being sufficient to enable them to overcome the electrical potential barriers presented by the nanoparticle interfaces.

**Figure 8:**
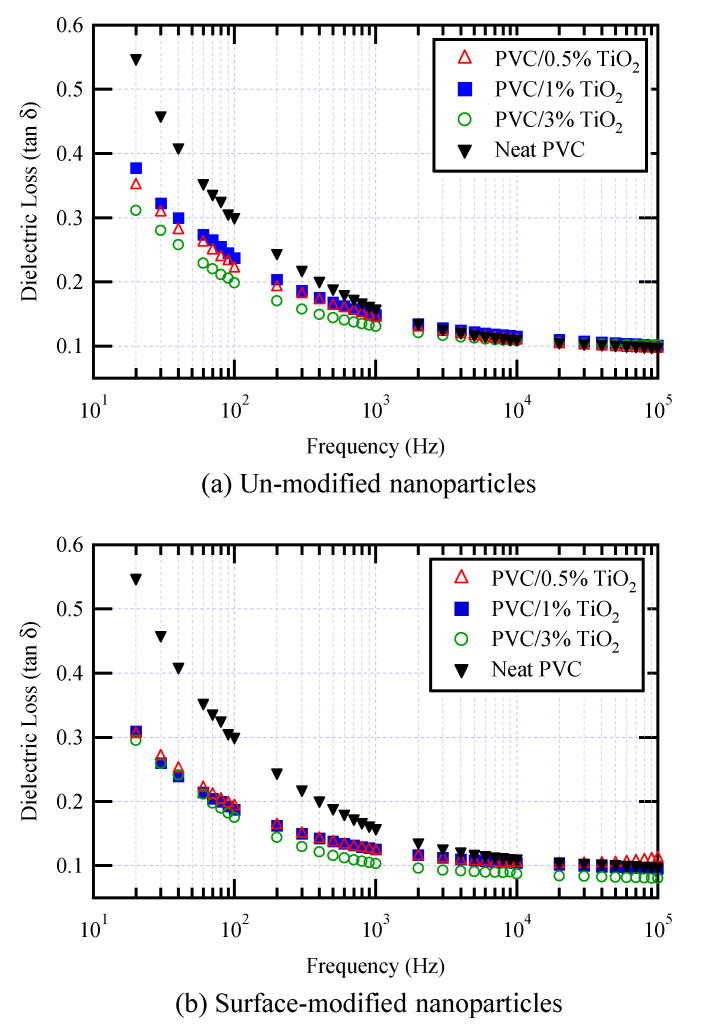
Dielectric loss of PVC/TiO_2_ nanocomposites; (**a**) un-modified nanoparticles and (**b**) surface-modified nanoparticles.

Few data on dielectric loss in nanocomposite insulation materials have been published. Figure 8 shows some results for PVC/TiO_2_ nanocomposites, with and without vinyl silane-based surface modification of the TiO_2_ nanoparticles [60]. Similar decreases in dielectric loss following the addition of SiO_2_ nanocomposites functionalized by amino silane to PVC have been observed [64], but only at frequencies below 1 kHz. LDPE/MgO nanocomposites also exhibited a lower dielectric loss than either base LDPE or LDPE microcomposites [94]. However, at frequencies below about 300 Hz, dielectric losses measured in LDPE/carbon black nanoparticles were considerably higher than those in LDPE [93].

### 3.6. Space Charge Profiles

Insulating materials in high voltage cables exhibit space charge accumulation, which can distort the electric field distribution within the insulation and possibly lead to breakdown [95]. Many studies have investigated the space charge profile in polymer nanocomposites used in cable insulation [96,97,98,99,100,101,102,103,104,105].

Several methods of measuring space charge profiles in insulators have been developed over the last 30 years [106,107,108]. Of these, the pulsed-electro-acoustic (PEA) method [109] and the thermal-step-method (TSM) [110] are frequently used. Space charge in the insulation of power cables can originate in charge injection from the central conductor and the semiconductor layer, and in ionization of residual additives and cross-linking by-products in the insulation. Homo-charges (with the same charge polarity as the injecting electrode) can decrease the electric field at and near the electrode/insulator interface, and therefore increase the electric field in the bulk of the insulator. In contrast, hetero-charges (with polarity opposite that of the injecting electrode) can increase the electric field at the electrode/insulator interface, and decrease the electric field in the bulk of the insulator.

LDPE and XLPE exhibit hetero-charge accumulation in the vicinity of the electrodes [96]. The measurements were made with the electric field applied. Hetero-charge concentration, especially at the cathode, increases with increasing applied field [42,96,99] and with increasing temperature gradient [101,111]. As stated above there are two main sources of space charge, namely injection from the electrodes and ionization of impurities, usually present throughout the bulk of the sample [42,99]. Injection from the electrodes usually leads to homo-charge accumulation near the electrodes, while ionization of impurities throughout the bulk and transport of charge carriers across the sample to the electrodes usually leads to hetero-charge accumulation near the electrodes.

The addition of nanoparticles to LDPE and XLPE may significantly reduce or suppress hetero-charge accumulation. Homo-charge suppression following nanoparticle addition is less pronounced than that of hetero-charge [42]. Factors such as nanoparticle weight fraction and grain size, sample thickness, and mechanical stressing may also be important. Space charge profiles in LDPE/MgO [42] and in LDPE/ZnO nanocomposites [99] have been studied at several nanoparticle weight fractions. In both types hetero-charge accumulation at the cathode was suppressed up to 1% nanoparticle loading; above this loading, hetero-charge accumulation near the cathode was observed. Hetero-charge accumulation was not observed at the anode, either for neat LDPE or for LDPE nanocomposites. In LDPE/ZnO nanocomposites homo-charge accumulation was also suppressed near both electrodes up to 0.1% nanoparticle loading [99]. To explain these results, it has been proposed [79] that a large concentration of charge carrier traps exists at the nanoparticle/polymer interfaces (the loose layer of the multi-core model) throughout the sample bulk. When such traps near an injecting electrode are occupied by charge carriers, they constitute a concentrated homo-charge layer which decreases the electric field at the electrode interface, thereby increasing the potential barrier to further charge injection and decreasing the accumulated homo-charge concentration. On the other hand, the deep traps introduced at the interface zones between fillers and polymer matrix [112] limit charge transport from the counter electrode (mainly anode in case of LDPE) through the sample bulk, leading to a suppression of hetero-charge accumulation. The higher the trap level density, the lower the charging current, and the slower the transport of charges injected from the electrode [99].

The size of nanoparticle clusters increases with increasing nanoparticles’ weight fraction. Such agglomeration increases again the hetero-charge density at the cathode similar to neat LDPE, presumably by reducing the number of traps at the nanoparticle/polymer interfaces within the sample bulk making again easy charge transport from the anode to the cathode and accumulation of hetero-charges at the cathode.

The effect of nanoparticle grain size on space charge accumulation in LDPE/MgO nanocomposites has been investigated using the PEA method [96]. The main observation was that the hetero-charge density near the electrodes decreased with the addition of MgO nanoparticles compared to the hetero-charge density observed in untreated LDPE, presumably due to reduced charge transport through the sample bulk as mentioned above. Homo-charge density for MgO nanoparticle sizes in the range 100–500 nm became greater than that for 30 nm, presumably because the height of the potential barrier between the electrodes and the LDPE bulk decreases with increasing nanoparticle size. Increasing nanoparticle size decreases the overall surface area at the interface zone between nanoparticles and polymer matrix. For homo-charges, this results in a decrease in the height of the potential barrier and causes further charge injection. For hetero-charges, this limits charge trapping and facilitates charge transport through the sample bulk causing an increase in hetero-charge accumulation similar to that occurred with nanoparticles agglomeration.

The influence of sample thickness on space charge formation in XLPE/SiO_2_ nanocomposites [101] and in LDPE/SiO_2_ nanocomposites has been compared with that in untreated XLPE and LDPE, at the same electric field strength. High hetero-charge concentrations were measured close to the cathode in all three untreated samples, but not in the nanocomposites, and small hetero-charge concentrations were measured close to the anode in both types of 0.1 mm thick samples. It was suggested [102] that charge carrier transport through the sample bulk is reduced in the nanocomposite sample due to the total number of deep traps provided by nanofillers along the path of charge transport from the anode to the cathode. The thicker the nanocomposite sample the larger number of deep traps and the lower the hetero-charge density. However, for all considered thicknesses in [102], hetero-charges were suppressed at all. A small amount of hetero-charge was observed close to the anode in a 0.1 mm thick nanocomposite sample, but not in 0.3 and 0.5 mm thick samples.

The effect of mechanical stretching on space charge formation in LDPE/MgO nanocomposites has been studied [70]. The elongation ratio normal to the electric field direction was 1.1. This ratio is defined as the ratio between final stretched length and initial un-stretched one [113]. It was suggested that the loose layer surrounding the nanoparticles, as envisaged in the multi-core model [79], increased in width as a result of the stretching, resulting in an increase in the concentration and depth of charge traps. The stretching may also have converted some shallow traps to deep traps, causing an increase in volume resistivity.

A bipolar charge transport (BCT) model has been used to simulate space charge accumulation in untreated polymers and nanocomposites. This model is consistent with an increase of hetero-charge accumulation at both electrodes with increasing electrode temperature difference observed in untreated XLPE [104], and with the observation in LDPE nanocomposites that increasing trap density reduces the electric field at the interface between the electrodes and the nanocomposite, due to the creation of a large barrier to charge injection [105].

It seems that the space charge profile for PVC nanocomposites is not available in the open literature and needs to be studied.

### 3.7. Partial Discharge and Treeing Resistance

**Figure 9:**
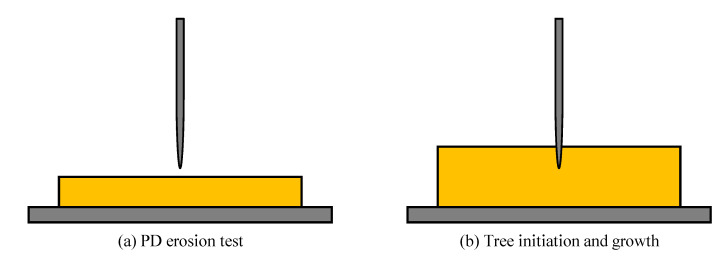
Electrode setup used for investigating PDs in polymer nanocomposites; (**a**) PD erosion test and (**b**) tree growth.

Partial discharge (PDs), and its impact on polymer dielectrics, have been attracted the interest of researchers for many years. This is because PD is one of the main degradation mechanisms [114], and also it can be used as a diagnostic indicator in polymer dielectrics [115,116,117,118,119]. PD is defined as localized discharge within the dielectric that partially bridges the insulation between two adjacent conductors. It has many types such as internal discharge, corona, and surface discharge. Many studies have been made on the resistance of nanocomposites based on polyethylene to electrical treeing [80,120,121], or water treeing [122]. It was found that dielectric nanocomposites effectively enhance the resistance against PD. Accordingly, several researchers investigated the enhancement of PD resistance for PE and PVC nanocomposites. For investigating PDs in polymer nanocomposites, a rod to plane electrode has been used with two different configurations as shown in Figure 9. The first configuration in Figure 9a is used for the PD erosion test, while, the second configuration in Figure 9b is used for testing tree initiation and growth.

**Figure 10:**
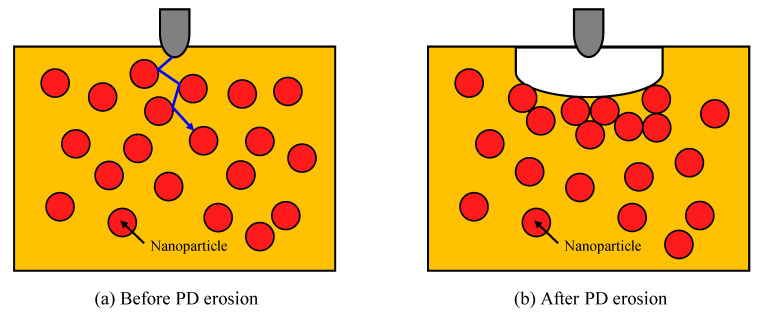
Mechanisms behind reducing PD erosion in polymer nanocomposites; (**a**) before PD erosion and (**b**) after PD erosion.

Tanaka et al. [120] investigated PD in LDPE/MgO nanocomposites with an applied voltage of 4 kV and a frequency of 720 Hz for a duration of 48 h. They found that the erosion depth decreased with increasing filler concentration. At 10% weight fraction of MgO the erosion depth was about 36% of its value in untreated LDPE. In [121], the PD inception voltage decreased slightly in LDPE/MgO nanocomposites at MgO loadings below 2%, and then increased at higher loadings. The PD detection sensitivity considered in [121] was 4 pico-columns (pC). It was suggested that the reduced erosion depth could be attributed to increasing resistance of exposed polymer surfaces to PD when they contain nanoparticles and to the multi-core morphology formed at polymer/nanofiller interfaces (bonded, bound, and loose layers). In addition, after PD is induced from the rod tip in a rod-plane electrode configuration, nanofillers around the tip are detached from the polymer matrix and accumulate on the sample surface, thereby providing additional PD resistance. The mechanism behind reducing PD erosion and enhanced PD resistance was explained in terms of the role of nanofillers in protecting the polymer matrix, and this is due to their strong PD resistance, Figure 10a. In addition, after PD erosion, nanofillers are separated from the polymer matrix and are grouped together on the surface resulting in an increase in PD resistance as shown in Figure 10b.

Studies of tree initiation and growth in LDPE/alumina nanocomposites were reported in [80]. The maximum suppression was obtained at 3% alumina weight fraction, when the average PD tree inception voltage was 20 kV_rms_, compared with 10 kV_rms_ for unfilled LDPE. Branch-type tree growth was found in unfilled LDPE, but bush-type in the nanocomposites, resulting in an increase in the density of tree branches and a reduction in branch length in nanocomposites. The authors suggested that the bush-type pattern in the nanocomposites could be due to the formation of charge traps at the nanoparticle surfaces, leading to the formation of secondary branches from the main tree branch. In LDPE/MgO nanocomposites the tree length decreased with increasing MgO loadings up to 2 wt %, but changed little at higher loadings [121].

Zhou et al. [122] found that TiO_2_ nanoparticles tended to fill voids in XLPE which otherwise would be filled with water. The distortion of the electric field in the volume surrounding a nanoparticle-filled void is much smaller than that in the volume surrounding a water-filled void, so that the breakdown strength is greater in the former case. Thus, inorganic nanoparticles effectively protect the XLPE chains against PD erosion.

For PVC nanocomposites, there are limited studies on PD activity [123,124]. For neat PVC, the inception voltage applied to a needle tip was 2 kV_rms_ and the average discharge magnitude was 589 pC. While, the inception voltage increased to 2.35 kV_rms_ and the average discharge magnitude decreased to 494 pC when using amino-treated PVC/TiO_2_ nanocomposites [124].

## 4. Suggested Future Work

In spite of many researches have investigated the breakdown strength and dielectric properties of PE and PVC nanocomposites, it is challenging to disperse nanoparticles homogeneously within the polymer matrix. This issue is critical for industrial-scale nanocomposites and has to be dealt in-depth in the future studies. The studies in this field can include developing advanced chemical and physical techniques capable to achieve homogeneous dispersion on the long-term operation of these materials. Additionally, it is desired to develop on-line techniques capable to observe the dispersion condition during real field operation of such materials.

Another future trend that can be beneficial for PE and PVC nanocomposites is to use special types of nanoparticles. The first promising type is porous nanoparticles that have embedded cavities in the nanosized scale. Porous nanoparticles could achieve lower permittivity for epoxy nanocomposites [125], but there are no available studies on the breakdown strength and dielectric properties of PE and PVC nanocomposites filled with such nanoparticles. The second promising type of nanoparticles is core-shell nanoparticles. A core-shell nanoparticle is composed of a nanoparticle in the core encapsulated by a thin shell of another material. Such structures can exhibit properties different than those of both materials. Using core-shell nanoparticles will aid in designing functional nanocomposites that can achieve multiple enhancements [126,127].

## 5. Conclusions

There are considerable discrepancies between the results reported by different authors for very similar nanodielectrics based on polyethylene and polyvinylchloride hosts. Some very general and tentative observations are as follows:

- Increases in breakdown strength have often been reported, and sometimes decreases, depending on the type and weight fraction of the nanoparticles.
- Permittivity also increases or decreases, depending on the type and size of the nanoparticles and on the coupling agent used for modification of their surfaces.
- DC conductivity and dissipation factor tend to decrease with the addition of nanoparticles.
- The addition of nanoparticles to polyethylene significantly suppresses hetero-charge accumulation observed at the cathode for neat LDPE; provided the nanoparticles are not agglomerated and have grain size less than about 100 nm. The enhancement is more pronounced in thicker samples.
- Nanoparticles tend to increase PD inception voltages and reduce surface erosion caused by PD. They also delay the onset of tree formation, as a function of applied voltage, and favor bush type trees.

## Figures and Tables

**Table 1:** AC breakdown strength of polymer nanocomposites based on PE and PVC.

Ref.	Polymer Nanocomposites	Mean Size (nm)	Concentrations	Best Concentration	Key Findings
[5]	HDPE/CaCO_3_	20–40	1, 2, and 4 wt %	2 wt %	Breakdown strength of HDPE + 1% g-maleic anhydride enhanced with 21% than the neat sample.
[19]	PVC/SiO_2_	20	1, 2.5, 5, and 7.5 wt %	5 wt %	Breakdown strength enhanced with 7% rather than the neat sample.
[43]	LLDPE/SiO_2_	12	2.5 wt %	2.5 wt %	Sample with treated octa-silica has higher breakdown strength than neat and/or untreated one.
[56]	LDPE/Al_2_O_3_	40	1.5, 3, 6, 12, and 15 wt %	1.5 wt %	Breakdown strength enhanced with 0.88% than neat, and the others decreased.
[57]	HDPE/clay	10	2, 6, 10, and 15 wt %	6 wt %	Breakdown strength enhanced with 15.4% rather than the neat sample.
[58]	HDPE/clay LDPE/clay	10	1, 5, and 10 wt %	10 wt %	Breakdown strength of HDPE/clay and LDPE/clay enhanced with 128.5% and 123.5% than the neat sample.
[58]	HDPE/SiO_2_ LDPE/SiO_2_	10	1, 5, and 10 wt %	1 wt %	The breakdown strength of HDPE/SiO_2_ and LDPE/SiO_2_ has the lowest reduction with 6.4% and 10.8% than the neat one.
[59]	LDPE/Al_2_O_3_	45	1 and 3 wt %	3 wt %	Breakdown strength enhanced with 13.5% rather than the neat sample.
[60]	PVC/TiO_2_	21	0.5, 1, 3, and 5 wt %	5 wt %	Breakdown strength enhanced with 10.9% for treated TiO_2_ and with 4.5% for un-treated one rather than a neat sample.
[61]	XLPE/SiO_2_	12	5 wt %	5 wt %	Breakdown strength enhanced with 2% for treated SiO_2_ and decreased by 5% for un-treated one rather than a neat sample.
[62]	LDPE/TiO_2_	Not specified	3 wt %	3 wt %	Breakdown strength enhanced with 9.2% for treated TiO_2_ and with 7.7% for un-treated one rather than a neat sample.
[63]	LDPE/POS	<500 µm	1 and 5 wt %	1 wt %	Breakdown strength enhanced with 7.2% for isooctyl-POS rather than the neat one.
[64]	PVC/SiO_2_	10–20	0.5, 1, 3, and 5 wt %	0.5 wt %	Breakdown strength enhanced with 14.1% for treated SiO_2_ and with 7.7% for un-treated one rather than a neat sample.
[65]	PVC/TiO_2_	21	0.5, 1, and 3 wt %	3 wt %	Breakdown strength enhanced with 8% for vinyl-treated TiO_2_ and with 3% for un-treated one rather than a neat sample.

**Table 2:** DC breakdown strength of polymer nanocomposites based on PE and PVC.

Ref.	Polymer Nanocomposites	Mean Size (nm)	Concentrations	Best Concentration	Key Findings
[42]	LDPE/MgO	50	0.1, 0.5, 1, 2, and 5 wt %	1 wt %	Breakdown strength enhanced with 9.6% rather than the neat sample.
[47]	XLPE/GO	<80	0.01 wt % (fGO-QWs)	0.01 wt %	Breakdown strength enhanced with 33.3% for functionalized graphene oxide quantum wells (fGO-QWs) than those of the neat XLPEs.
[54]	PVC/ZnO	28–41.5	0.3, 0.5, 0.7, 1, 3, and 5 wt %	0.5 wt %	Breakdown strength enhanced with 45% rather than the neat sample.
[60]	PVC/TiO_2_	21	0.5, 1, 3, and 5 wt %	5 wt %	Breakdown strength enhanced with 14.2% for treated TiO_2_ and with 4.8% for un-treated one rather than a neat sample.
[64]	PVC/SiO_2_	10–20	0.5, 1, 3, and 5 wt %	0.5 wt %	Breakdown strength enhanced with 16.3% for treated SiO_2_ and with 8.8% for un-treated one rather than a neat sample.
[65]	PVC/TiO_2_	21	0.5, 1, and 3 wt %	3 wt %	Breakdown strength enhanced with 10% for vinyl-treated TiO_2_ and with 4.5% for un-treated one rather than a neat sample.
[67]	LDPE/Al_2_O_3_	30 ± 5	0.1, 0.5, 1, 2, 5 and 10 wt %	0.5 wt %	Breakdown strength enhanced with 35% rather than the neat sample.
[68]	LDPE/MgO	10	0.2, 0.5, 1, 2, 5 and 10 wt %	2 wt %	Breakdown strength enhanced with 46% rather than the neat sample.
[69]	LDPE/Al_2_O_3_	50–100	0.1, 0.2, 0.5, and 1 wt %	1 wt %	The treatment of nanoparticles does not change breakdown mechanisms of the composites rather than the neat sample.
[70]	LDPE/MgO	40	1, 3, and 5 wt %	1 wt %	Breakdown strength enhanced with 42.5% rather than the neat sample.
[71]	PVC/CaCO_3_	10–30	2.5, 5, and 7.5 wt %	5 wt %	Breakdown strength enhanced with 59.5% rather than the neat sample.
[71]	PVC/SiO_2_	10–30	2.5, 5, and 7.5 wt %	5 wt %	Breakdown strength enhanced with 48.8% rather than the neat sample.
[72]	PE/SiO_2_	10–20	2, 5, and 10 wt %	5 wt %	The lowest decrease in breakdown strength is 9% for treated SiO_2_ and all other samples including un-treated ones are lower than a neat sample.
[73]	XLPE/ZnO	20 & 200	1, 3, and 5 wt %	3 wt %	Breakdown strength enhanced with 9% (for ZnO = 200 nm) rather than the small size of ZnO samples and/or neat one.

**Table 3:** Impulse breakdown strength of polymer nanocomposites based on PE and PVC.

Ref.	Polymer Nanocomposites	Mean Size (nm)	Concentrations	Best Concentration	Key Findings
[68]	LDPE/MgO	10	1, 2, 5, and 10 wt %	2 wt %	Impulse wave; front duration 1.2 µs, and one shot impulse 800 kV/mm. Breakdown strength enhanced with 21% rather than the neat sample.
[76]	LDPE/MgO	50	1, 5 and 10 wt %	1 wt %	Impulse wave; three shot (-ve) impulse each step of 2 kV. Breakdown strength slightly enhanced with 4% rather than the neat sample.
[77]	LDPE/SiO_2_	30 ± 5	0.1, 0.5, 1, 2, and 5 wt %	1 wt %	Impulse wave; 1.2/50 µs, and one shot impulse 30 kV, then increased in steps of 1.5 kV/min. Breakdown strength enhanced with 10% rather than the neat sample.
[78]	LDPE/Al_2_O_3_	<100	0.5, 1, 5 and 10 wt %	0.5 wt %	Impulse wave; 1.2/50 μs, and one shot impulse 20 kV, then increased in steps of 2 kV/min. Breakdown strength enhanced with ~30% rather than the neat sample.

**Table 4:** Dielectric properties of polymer nanocomposites based on PE and PVC.

Ref.	Polymer Nanocomposites	Mean Size (nm)	Concentrations	Best Concentration	Key Findings
[60]	PVC/TiO_2_	21	0.5, 1, 3, and 5 wt %	3 wt %	Frequency range: 20–10^6^ Hz. The maximum reduction in permittivity was about 43% and dielectric loss about 41% for treated TiO_2_ rather than a neat sample at 50 Hz.
[64]	PVC/SiO_2_	10–20	0.5, 1, 3, and 5 wt %	0.5 wt %	Frequency range: 20–10^6^ Hz. The maximum reduction in permittivity was about 25% and dielectric loss about 39% for treated SiO_2_ rather than a neat sample at 50 Hz.
[65]	PVC/TiO_2_	21	0.5, 1, and 3 wt %	3 wt %	Frequency range: 20–10^6^ Hz. The maximum reduction in permittivity and dielectric loss are 43% and 41% for vinyl-treated TiO_2_, while equal 22% and 27% for amino-treated one, respectively, rather than a neat sample at 50 Hz.
[67]	LDPE/Al_2_O_3_	30 ± 5	0.1, 0.5, 1, 2, 5 and 10 wt %	0.5 wt %	The highest DC resistivity increase by a factor of about 10 compared with that of neat LDPE sample.
[69]	LDPE/Al_2_O_3_	50–100	0.1, 0.2, 0.5, and 1 wt %	1 wt %	Frequency range: 10^2^–10^6^ Hz. The maximum reduction in permittivity at 600 Hz was about 19.8% for treated Al_2_O_3_, and the DC resistivity increased about three times that of neat LDPE.
[88]	LDPE/GO	Thick = 1 nm, lateral dimension 0.5–5 μm	0.015 vol% coated	0.015 vol% coated	Frequency range: 10^−1^–10^5^ Hz. The permittivity slightly increased with thermal treated GO at all frequencies. Otherwise, below 4 kV/mm the nanocomposites showed lower conductivity than neat LDPE.
[89]	LDPE/ MWCNT	20–30	0.02, 0.04, 0.08, and 0.1 vol%	0.02 vol%	Frequency range: 10^2^–10^6^ Hz. Permittivity increases to 100 compared to 2.2 for neat LDPE over 0.08 vol% at 100 Hz. Also, the minimum increase in conductivity happens for 0.02 vol%.
[91]	LDPE/MgO	10–20	0.1, 1, 3, 6, and 9 wt %	3 wt %	The conduction current is significantly dropped up to three orders in magnitude and DC conductivity decreased by 104% rather than a neat sample at 60 °C.
[92]	LDPE/Al_2_O_3_	50	1, 3, 5, and 10 wt %	1 wt %	The greatest reduction in DC conductivity happens by two orders of magnitude.
[93]	LDPE/CB	30	0.01, 0.03, 0.06, and 0.09 wt %	0.01 wt %	Frequency range: 1–10^5^ Hz. Permittivity, dielectric loss, and DC resistivity are slightly increased by 0.5%, 1%, and 5%, respectively compared with a neat LDPE at 50 Hz.

## Data Availability

The data presented in this study are available on request from the corresponding author.
